# Awareness and Perception of Anti-obesity Medications Among Al-Ahsaa, Riyadh, and Hail in Saudi Arabia Populations

**DOI:** 10.7759/cureus.40425

**Published:** 2023-06-14

**Authors:** Ebtehaj S Almughais, Manar H Alshehri, Munirah Alsatti, Amani Almatar, Fatima H Albladi, Heba H Almomatin, Nourah M Alshammari, Rozan Alshammari

**Affiliations:** 1 Family and Community Medicine, University of Hail College of Medicine, Hail, SAU; 2 College of Medicine, University of Hail College of Medicine, Hail, SAU

**Keywords:** exercise, awareness, weight loss, medications, obesity

## Abstract

Introduction: Obesity is the most prevalent medical disease afflicting low-, middle-, and high-income nations. Hence, the use of anti-obesity drugs is gaining popularity as an adjuvant treatment for this medical condition, along with diet and lifestyle modifications. Different medications have been approved for the treatment of obesity. So, it is of the utmost importance to know the community's perception and awareness on that matter.

Methods: An analytical, cross-sectional study design was adopted for this study. The data was collected by distributing an electronic questionnaire to adult males and females aged between 18 and 60 years living in Hail, Riyadh, and Al-Ahsaa, Saudi Arabia. The questionnaire consisted of three sections. The first section dealt with demographic data; the second section included items for measuring the awareness and general perception of anti-obesity medications; and the last section included one item used to recognize the reasons for refusing to take anti-obesity medications.

Results: A total of 1073 participants from Al-Ahsaa, Hail, and Riyadh completed the questionnaire. Out of the total, 55.6% had an overall good awareness level about anti-obesity medications. Regarding information about the drugs, 77.6% think that there is a certain body mass index (BMI) that allows the use of these medications; 31.4% reported a BMI of > 40. In addition, most participants think that these drugs can make them lose 3-8 kg per year. However, 69.3% and 64.5% think that anti-obesity drugs increase the risk of pancreatitis and thyroid tumors, respectively. Lastly, factors that are associated with a good overall awareness level are: females, participants living in Riyadh, post-graduate degrees, and work in the health care field.

Conclusion: The overall awareness of anti-obesity drugs was good, particularly in Riyadh, the kingdom's capital. However, the majority of the individuals who had low awareness of these medications would not take them if their doctors prescribed them, mainly due to their concern about the treatment's side effects.

## Introduction

The WHO defines obesity as having a BMI of 30 kg/m^2^ (class 1) or more [1]. The prevalence of obesity has increased by 27.5% in adults and 47.1% in children during the last three decades [2]. Obesity arises in the background of the complex interaction and regulation of calorie utilization, appetite, exercise, and socioeconomic status in association with the surrounding environmental factors as well as the underlying hereditary factors [3]. A report by WHO revealed that in 2016, 13% of adults over the age of 18 were obese [4]. Nonetheless, according to the Global Burden of Diseases (GBD) 2019 report, the Eastern Mediterranean Region (EMR) has the greatest rate of age-related obesity mortality [5]. Additionally, a recent study showed that Arab countries have one of the highest rates of obesity, with an estimation of 66%-75% of adults and 25%-40% of children in the Arab region being either overweight or obese [6]. Also, another study conducted in Riyadh, Kingdom of Saudi Arabia, showed that 82% of participants were overweight or obese [7].

Therefore, obesity reduction has the ability to lessen the financial and social strain on the healthcare system while also enhancing patient quality of life [8]. The lifestyle modification approach is the foundation of weight loss. Besides this, if this approach does not lead to satisfactory weight loss, emerging approaches, including pharmacotherapy, medical devices, and/or bariatric surgery, may be considered [9]. Pharmacotherapy has advanced in recent years, and many FDA-approved drugs could be used. Clinically, the choice and long-term use of drugs can be evaluated based on their tolerability and safety [10]. Semaglutide, liraglutide, phentermine-topiramate, orlistat, and naltrexone-bupropion are the five drugs approved to treat obesity. When compared to a placebo, semaglutide caused the greatest percentage of weight loss (10% body weight loss): 75.3% with semaglutide vs. 27.0% with the latter [11].

## Materials and methods

An analytical, community-based, cross-sectional study design was adopted for this research. The population of this study consists of adult males and females living in Hail, Riyadh, and Al-Ahsaa, Saudi Arabia. The inclusion criteria were people of both sexes, aged between 18 and 60, who live in Hail, Riyadh, and Al-Ahsaa, Saudi Arabia. And exclusion criteria were males and females aged under 18 or over 60 years old. In addition, anyone who lives in a city other than Hail, Riyadh, or Al-Ahsaa was excluded.

Following a thorough literature review and due to the lack of similar studies, a self-made, 16-item questionnaire was developed. The questionnaire was presented to two consultants, who approved its validity. It is translated into Arabic, the native language of the participants. The ethical approval (number 317) for this research was obtained from the Institutional Ethical Committee at the University of Hail on September 26, 2022.

All participants were kindly asked to voluntarily complete the electronic questionnaire that was randomly sent to the population of the selected cities, with consent at the beginning of the questionnaire, explaining the study and its objectives.

The first section of the questionnaire dealt with the demographic data of the participants. This section was used to ensure the study participants' inclusion criteria. The second section includes items to measure awareness and general perception of anti-obesity medications. These questions were used to assess the indication and eligibility for the use of anti-obesity medications, as well as the mode of action, side effects, effectiveness, and complications of the medications. The last section included one item used to identify the reasons for refusing to take anti-obesity medications.

Data analysis

The data were collected, reviewed, and then fed to Statistical Package for Social Sciences (SPSS), version 21 (IBM Corp., Armonk, NY). All statistical methods used were two-tailed with an alpha level of 0.05 considering significance if the P value is less than or equal to 0.05. Overall awareness level regarding anti-obesity drugs was assessed by summing up discrete scores for correct awareness of different items. The overall awareness score was categorized as a poor level if the participants' score was less than 60% of the overall score and a good level of awareness was considered if the participant’s score was 60% or more of the overall score. Descriptive analysis was done by prescribing frequency distribution and percentage for study variables including participants' personal data, residence, and work field. Also, awareness regarding anti-obesity drugs and their perception were tabulated for different domains and overall awareness levels and sources of information were graphed. Cross tabulation for showing factors associated with study participants' awareness of anti-obesity drugs and the relation between participants' awareness and their perception of the drugs was carried out with Pearson Chi-square test for significance and exact probability test if there were small frequency distributions.

## Results

A total of 1073 participants completed the study questionnaire, 425 (39.6%) were from Al-Ahsaa, 404 (37.7%) from Riyadh, and 244 (22.7%0 from Hail. Participants' ages ranged from 18 to more than 60 years with mean age of 32.4 ± 13.5 years old. A total of 594 (55.4%) were females. As for education level, 267 (24.9%) had a secondary level of education/below secondary level, 346 (32.2%) were university students, and 460 (42.9%) were university graduate/post-graduate degrees. Exactly 181 (16.9%) worked in the healthcare field (Table 1).

**Table 1:** Personal data of study participants, Saudi Arabia

Personal data	No	%
Residence		
Al-Ahsaa	425	39.6%
Hail	244	22.7%
Riyadh	404	37.7%
Age in years		
< 20	59	5.5%
20-39	725	67.6%
40-59	265	24.7%
60+	24	2.2%
Gender		
Male	479	44.6%
Female	594	55.4%
Educational level		
Secondary / below	267	24.9%
University student	346	32.2%
University graduate	400	37.3%
Post-graduate degree	60	5.6%
Work at health care field		
Yes	181	16.9%
No	892	83.1%

Table 2 shows that an exact percentage of 37.1% of the study participants had information about anti-obesity drugs, 76% refused that anyone can use anti-obesity drugs to lose weight, and 77.6% think there is a certain body mass index (BMI) that allows the use of anti-obesity drugs. A total of 29.7% reported that BMI > 30 (obesity class I) would allow the use of anti-obesity drugs among healthy persons, while 24% reported BMI > 35 (obesity class II) and 31.4% reported BMI > 40 (obesity class III). As for the mechanism of action, 35.4% reported decreasing appetite, 16.6% reported promoting satiety, 13.9% reported reducing fat absorption, and 56.3% reported all. A total of 85.5% think that these drugs can help you lose 3-8 kg of weight per year. Considering side effects, the most reported included nausea and vomiting (71%), depression and mood swings (61.2%), diarrhea and constipation (51.6%), and drowsiness and headache (51.4%). A total of 69.3% think that anti-obesity drugs may increase the risk of pancreatitis, 64.5% think they may increase the risk of thyroid tumors, and only 22.6% incorrectly reported that anti-obesity drugs are more effective without diet and exercise.

**Table 2:** Awareness of anti-obesity medications among Riyadh, Hail and Al-Ahsaa populations, Saudi Arabia

Items to measure the awareness of anti-obesity medications:	No	%
Do you have any information about anti-obesity drugs before?		
Yes	398	37.1%
No	675	62.9%
Do you think anyone can use anti-obesity drugs to lose weight?		
Yes	257	24.0%
No	816	76.0%
Do you think there is a certain body mass index (BMI) that allows the use of anti-obesity drugs?		
Yes	833	77.6%
No	240	22.4%
What percentage of body mass index (BMI) would allow the use of these drugs in healthy subjects?		
BMI > 25	160	14.9%
BMI > 30	319	29.7%
BMI > 35	257	24.0%
BMI > 40	337	31.4%
Mechanism of action of anti-obesity drugs on the human body?		
Decrease appetite	380	35.4%
Promote satiety	178	16.6%
Reduce fat absorption	149	13.9%
All of the above	604	56.3%
Do you think that these drugs can lose 3 - 8 kg of weight / year?		
Yes	917	85.5%
No	156	14.5%
Side effects of anti-obesity medications		
Nausea & vomiting	762	71.0%
Diarrhea & constipation	554	51.6%
Depression & mood swings	657	61.2%
Drowsiness / headache	552	51.4%
Do you think that anti-obesity drugs may increase the risk of pancreatitis?		
Yes	744	69.3%
No	329	30.7%
Do you think that anti-obesity drugs may increase the risk of thyroid tumors?		
Yes	692	64.5%
No	381	35.5%
Do you think anti-obesity drugs are more effective without diet and exercise?		
Yes	242	22.6%
No	831	77.4%

**Figure 1:**
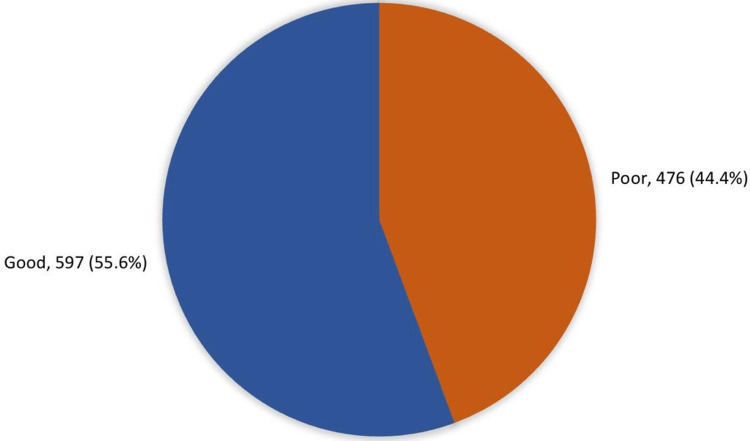
Overall awareness level of anti-obesity medications among Riyadh, Hail and Al-Ahsaa populations, Saudi Arabia

Figure 1 presents the overall awareness level of anti-obesity medications, which shows that 597 (55.6%) had an overall good awareness level regarding the drugs, while 476 (44.4%) had a poor awareness level.

**Figure 2:**
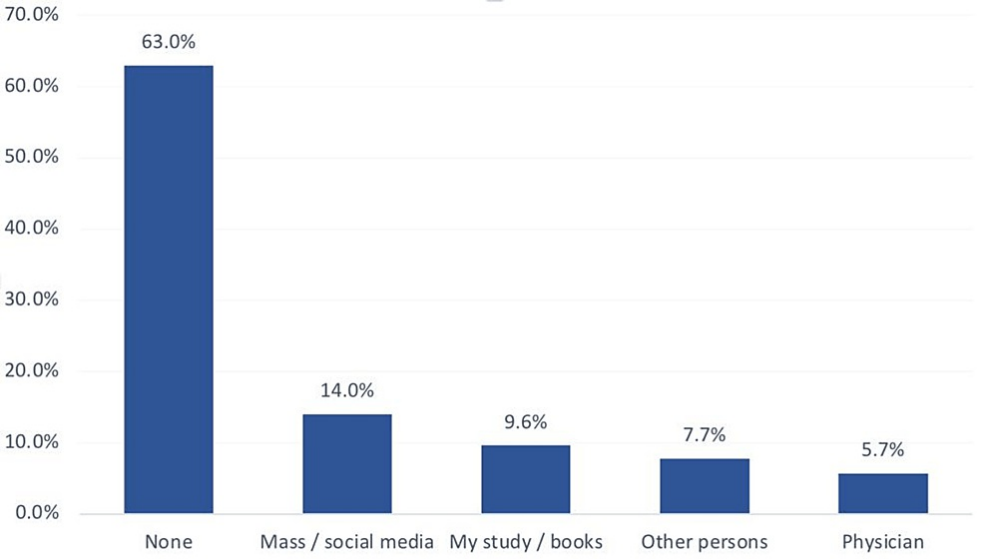
Source of participants' information regarding anti-obesity medications among Riyadh, Hail and Al-Ahsaa Populations, Saudi Arabia

We found that the most reported sources were mass and social media (14%), studies and books (9.6%), other persons (7.7%), and physicians (5.7%), while most of them had no specific source (63%) (Figure 2).

Our results show that exactly 724 (67.5%) participants reported that they could use anti-obesity drugs if advised by their physicians. The most reported reasons for refusing, among others, were fear of side effects and complications (31.5%), preferring diet/exercises (31.5%), don’t prefer drug intake (20.1%), they were not obese so no need to use (11.7%), and others (Table 3).

**Table 3:** Perception of anti-obesity medications among Riyadh, Hail and Al-Ahsaa populations, Saudi Arabia

Perception	N	%
If a physician advised you to use anti-obesity drugs, would you use them?		
Yes	724	67.5%
No	349	32.5%
If not, why?		
Side effects/complications	110	31.5%
Prefer diet/exercises	110	31.5%
Don't prefer/trust drugs	70	20.1%
Not obese/no need	41	11.7%
Other natural/safe alternatives	10	2.9%
Ineffective/lack of information	8	2.3%

We found in our study that 61.9% of participants from Riyadh had an overall good awareness level versus 48.8% of Hail participants, with recorded statistical significance (P =.003). Also, 61.1% of female participants had good awareness compared to 48.9% of males (P=.001). Good awareness regarding the drugs was detected among 78.3% of participants with post-graduate degrees compared to 47.2% of others with a low level of education (P=.001). A total of 65.7% of those who work in the healthcare field had an overall good awareness in comparison to 53.6% of others (P=.003). Also, 89.3% of participants who gained information from the study had an overall good awareness versus 42.2% of others with no specific source (P=.001) (Table 4).

**Table 4:** Factors associated with participants' awareness level regarding anti-obesity drugs P: Pearson X2 test     $: Exact probability test     * P < 0.05 (significant)

Factors	Overall awareness level				p-value
	Poor		Good		
	No	%	No	%	
Residence					.003*
Al-Ahsaa	197	46.4%	228	53.6%	
Hail	125	51.2%	119	48.8%	
Riyadh	154	38.1%	250	61.9%	
Age in years					.208
< 18	25	42.4%	34	57.6%	
18-39	309	42.6%	416	57.4%	
40-59	128	48.3%	137	51.7%	
60+	14	58.3%	10	41.7%	
Gender					.001*
Male	245	51.1%	234	48.9%	
Female	231	38.9%	363	61.1%	
Educational level					.001*
Secondary / below	141	52.8%	126	47.2%	
University student	130	37.6%	216	62.4%	
University graduate	192	48.0%	208	52.0%	
Post-graduate degree	13	21.7%	47	78.3%	
Work at health care field					.003*
Yes	62	34.3%	119	65.7%	
No	414	46.4%	478	53.6%	
Source of information					.001*^$^
None	390	57.8%	285	42.2%	
Physician	22	36.1%	39	63.9%	
Mass / social media	28	18.7%	122	81.3%	
My study / books	11	10.7%	92	89.3%	
Other persons	25	30.1%	58	69.9%	

Table 5 reveals that 65.3% of those with good awareness agreed to use prescribed ant-obesity drugs versus 70.2% of others with poor awareness, with no statistical significance. A total of 35.3% of others with good awareness refused due to side effects and complications, while 30.4% of them refused due to preference for diet and exercise, versus 26.1% and 33.1% of those with poor awareness, respectively (P =.012).

**Table 5:** Association between participants' awareness level and their perception regarding anti-obesity drugs $: Exact probability test     * P < 0.05 (significant)

Perception	Overall awareness level				p-value
	Poor		Good		
	No	%	No	%	
If a physician advised you to use anti-obesity drugs, would you use them?					.093
Yes	334	70.2%	390	65.3%	
No	142	29.8%	207	34.7%	
If not, why?					.012*^$^
Side effects/complications	37	26.1%	73	35.3%	
Prefer diet/exercises	47	33.1%	63	30.4%	
Not obese/no need	26	18.3%	15	7.2%	
Don't prefer/trust drugs	26	18.3%	44	21.3%	
Other natural/safe alternatives	5	3.5%	5	2.4%	
Ineffective/lack of information	1	.7%	7	3.4%	

## Discussion

Our results indicated that the age group with the most responses was 20-39. According to a study conducted in Makkah, Saudi Arabia, the same age group provided the most responses [12]. Additionally, we discovered that there were more female participants than male participants, which was inconsistent with the results of the study conducted in Makkah [12]. This might be due to the fact that obesity is predicted to increase among females, and there is a need to learn more about weight loss medications [5]. As for education level, the majority of respondents were university graduates, followed by university students. In a study published in Medina, Saudi Arabia, most of the participants were university graduates or higher [13]. This indicates that education level plays a role in overall knowledge. Healthcare workers' participation was low compared with other participants.

We found in our study that the participants from Riyadh had an overall good awareness level compared to the participants from Hail, who had the lowest knowledge. This can be attributed to the fact that Riyadh is the capital of Saudi Arabia, it is more civilized, and public awareness, in general, is better than that of smaller communities like Hail. Additionally, the female participants had better awareness compared to the males. Also, in other research, females know more about anti-obesity medication than males [12]. This may indicate that the increase in women's knowledge about obesity and treatment methods is perhaps due to the fact that women have higher rates of obesity in Saudi Arabia than men [14]. In our study, we found that there was good awareness regarding the drugs among the participants with a post-graduate degree in comparison to others with a low level of education. Similarly, another study demonstrated that a higher educational level was significantly correlated with improved knowledge, attitudes, and practices [13]. According to our study, we found those who work in the healthcare field had an overall good level of awareness in comparison to others. This may be explained by the fact that healthcare workers generally have greater knowledge of health information and work experience than other respondents. Also, we found the participants who gained information from the study had an overall good awareness compared to others with no specific source, whereas in other research in Iraq, they got information and knowledge from social media or a physician [15].

High BMI prevalence has increased among Saudis, particularly among middle-aged individuals [16]. Most of the respondents claimed to have little information about anti-obesity medications. Despite this, they showed a good awareness of the indications for using anti-obesity medications in general. However, there is a knowledge gap regarding the BMI percentage that is eligible for these drugs. A study published in Madina, Saudi Arabia, found that participants with low educational levels and a high BMI had poor understanding and attitudes concerning obesity [13]. Another study conducted in the United States discovered that a high BMI is generally associated with low educational and socioeconomic levels [17]. Such factors are likely to be associated with low awareness about obesity management and eligibility for anti-obesity medications. To reduce this misconception, a community awareness campaign about medications for obesity is required.

Several United States Food and Drug Administration (US FDA)-approved anti-obesity medications work through different mechanisms, so each medication has a unique set of side effects [18]. According to the current study, participants are aware of anti-obesity drugs and their various effects on the human body. Anti-obesity medications, according to most respondents, may raise the risk of pancreatitis and thyroid cancer. This knowledge demonstrates that there is overall good knowledge, but that is regarded as insufficient when discussing each medication and its side effects. This could be attributable to the limited use of these medications, as well as the desire to adopt a lifestyle weight loss approach instead of drugs, as stated in this study and consistent with others [12,13]. Despite this preference, participants believe that anti-obesity medications are effective. In contrast to the Singapore study, their participants were doubtful regarding the effectiveness and efficacy of obesity medications [19].

The majority of respondents stated that; they could utilize anti-obesity medications if recommended by their doctors. This conclusion is consistent with a prior study from the USA, which found that 75% of primary care patients would take weight-loss drugs daily on the advice of their doctor [20].

Respondents with high awareness (65.3%) accepted to use prescription anti-obesity medicines, whereas those with low knowledge (70.2%) did not. Respondents who declined to use anti-obesity medication expressed concerns about side effects and complications. This finding was consistent with previous studies [21,22].

Another factor behind respondents' reluctance to take anti-obesity medications was their preference for modifications to their lifestyles that would assist them in losing weight. The fact that they preferred not to take medication and that they were not obese were further considerations.

## Conclusions

The overall awareness of anti-obesity drugs was good, particularly in Riyadh, the kingdom's capital, which was to be expected. However, the majority of the individuals who had low awareness of these medications would not take them if their doctors prescribed them, mainly owing to their concern about the treatment's side effects. Therefore, it is urged and recommended that the Ministry of Health develop and concentrate on community-based educational programs and campaigns on anti-obesity medications to boost the perception of these medications and improve the management of obesity.

## References

[1] (2023). A healthy lifestyle - WHO recommendations. https://www.who.int/europe/news-room/fact-sheets/item/a-healthy-lifestyle---who-recommendations.

[2] Apovian CM (2016). Obesity: definition, comorbidities, causes, and burden. Am J Manag Care.

[3] Lin X, Li H (2021). Obesity: epidemiology, pathophysiology, and therapeutics. Front Endocrinol (Lausanne).

[4] (2023). WHO: Obesity and overweight. https://www.who.int/news-room/fact-sheets/detail/obesity-and-overweight.

[5] Chong B, Jayabaskaran J, Kong G (2023). Trends and predictions of malnutrition and obesity in 204 countries and territories: an analysis of the Global Burden of Disease Study 2019. EClinicalMedicine.

[6] Rahim HF, Sibai A, Khader Y (2014). Non-communicable diseases in the Arab world. Lancet.

[7] Al-Haqwi A, Al-Nasir M, Ahmad N, Masaudi E, Alotaibi SS, Hamad B (2015). Obesity and overweight in a major family practice center, central region, Saudi Arabia. Saudi Journal of Obesity.

[8] Bragg R, Crannage E (2016). Review of pharmacotherapy options for the management of obesity. J Am Assoc Nurse Pract.

[9] Kahan S (2016). Overweight and obesity management strategies. Am J Manag Care.

[10] Gadde KM, Pritham Raj Y (2017). Pharmacotherapy of obesity: clinical trials to clinical practice. Curr Diab Rep.

[11] Idrees Z, Cancarevic I, Huang L (2022). FDA-approved pharmacotherapy for weight loss over the last decade. Cureus.

[12] Sharaf SE, Al-shalabi BT, Althani GF (2021). Obesity self-management: knowledge, attitude, practice, and pharmaceutical use among healthy obese individuals in Saudi Arabia. Int J Fam Community Med.

[13] Alhawiti RM (2021). Knowledge, attitude, and practice about obesity among adults (18-45 years) in primary health care in Medina, KSA 2019. Int J Med Dev Countries.

[14] Salem V, AlHusseini N, Abdul Razack HI, Naoum A, Sims OT, Alqahtani SA (2022). Prevalence, risk factors, and interventions for obesity in Saudi Arabia: a systematic review. Obes Rev.

[15] Jasim UT, Ebrahim SM, Ajimi ES, Hassan ZS (2023). Knowledge and use of anti-obesity medications among students at Basrah University. Eurasian Med Res Periodicals.

[16] Wahabi H, Fayed AA, Shata Z (2023). The impact of age, gender, temporality, and geographical region on the prevalence of obesity and overweight in Saudi Arabia: scope of evidence. Healthcare (Basel).

[17] Lyu B, Chang AR, Inker LA, Selvin E, Grams ME, Shin JI (2022). Socioeconomic status and use of obesogenic and anti-obesity medications in the United States: a population-based study. Lancet Reg Health Am.

[18] Kumar RB, Srivastava G, Reid TJ, Aronne LJ (2021). Understanding the pathophysiologic pathways that underlie obesity and options for treatment. Expert Rev Endocrinol Metab.

[19] Lee PC, Ganguly S, Tan HC (2019). Attitudes and perceptions of the general public on obesity and its treatment options in Singapore. Obes Res Clin Pract.

[20] Wee CC, Sugai E, Aliberti G, Chiodi S (2017). Willingness to take weight loss medication among obese primary care patients. J Clin Outcomes Manag.

[21] Cook NS, Tripathi P, Weiss O, Walda S, George AT, Bushell A (2019). Patient needs, perceptions, and attitudinal drivers associated with obesity: a qualitative online bulletin board study. Adv Ther.

[22] Bessesen DH, Van Gaal LF (2018). Progress and challenges in anti-obesity pharmacotherapy. Lancet Diabetes Endocrinol.

